# Advances in Foodborne Pathogen Detection: From Conventional Confirmation to Integrated and Intelligent Platforms

**DOI:** 10.3390/foods15111983

**Published:** 2026-06-03

**Authors:** Xiang Pan, Xiong Ding

**Affiliations:** 1 Key Laboratory of Environmental Medicine and Engineering, Ministry of Education, Department of Nutrition and Food Hygiene, School of Public Health, Southeast University, Nanjing 210009, China; 2 Suzhou Research Institute, Southeast University, Suzhou 215123, China

**Keywords:** foodborne pathogens, rapid detection, nucleic acid amplification, biosensors, CRISPR-Cas, microfluidics, food safety monitoring

## Abstract

Foodborne pathogens pose a major challenge for public health, food safety regulation, and industrial quality control. Effective surveillance, outbreak tracing, and early warning for foodborne microbial contamination require rapid, reliable detection methods. Conventional culture-based methods are still essential for regulatory confirmation since they recover viable isolates and support downstream verification. However, their long turnaround time, labor-intensive procedures, and limited throughput restrict their use in rapid screening and on-site testing. In recent years, immunological assays, nucleic acid amplification and recognition methods, biosensors, microfluidic systems, CRISPR-Cas platforms, mass spectrometry, sequencing technologies, and artificial intelligence-assisted analysis have expanded the detection toolbox. These methods improve speed, sensitivity, portability, and multiplexing capacity, but their performance still depends on food-matrix properties, sample pretreatment, and application conditions. This review compares representative methods in terms of analytical principle, sample pretreatment, sensitivity, specificity, assay time, viable-cell discrimination, field applicability, and standardization potential. In our opinion, culture-based methods are central for confirmation, while emerging technologies are better suited for rapid screening, integrated analysis, and point-of-need testing. Nevertheless, matrix interference, limited validation in naturally contaminated samples, insufficient viable/dead-cell discrimination, and weak cross-platform consistency remain key barriers to routine use.

## 1. Introduction

Food safety remains a major public health issue. Foodborne pathogens continue to cause illness, hospitalization, death, and economic loss worldwide. This problem has become more difficult to control as modern food systems have increased in scale and complexity. Industrial processing, global supply chains, and cold-chain logistics allow food products to pass through multiple stages before consumption. Contamination or cross-contamination may occur during raw material sourcing, processing, storage, transportation, or retail. These stages increase the diversity of contamination sources and make transmission routes harder to trace [1]. Such risks are especially relevant to meat products, aquatic products, dairy products, fruits and vegetables, ready-to-eat foods, and infant formula.

The World Health Organization (WHO) estimates that contaminated food causes about 600 million illnesses and 420,000 deaths each year. Unsafe food also leads to an annual loss of approximately 33 million disability-adjusted life years. In low- and middle-income countries, foodborne diseases cause an estimated 110 billion US dollars in productivity losses and healthcare costs each year [2,3]. Therefore, rapid, accurate, robust detection methods compatible with complex food matrices are urgently demanded for foodborne disease control, enterprise quality management, and regulatory early warning.

The burden is not evenly distributed across pathogen groups. WHO estimates showed that, among foodborne diarrheal disease agents, norovirus caused approximately 125 million illnesses and 35,000 deaths, while *Campylobacter* spp. caused approximately 96 million illnesses [4]. The same series also reported that hepatitis A virus caused approximately 14 million foodborne illnesses and 28,000 deaths [5], and that non-typhoidal *Salmonella enterica* contributed one of the highest disease burdens, with about 4.07 million disability-adjusted life years [4]. Foodborne parasites, such as *Taenia solium*, *Giardia* spp., and foodborne trematodes, also contributed substantially in specific regions or age groups [6]. These differences provide additional context for the need to develop rapid, sensitive, and matrix-compatible detection technologies. Although this review mainly focuses on bacterial foodborne pathogens, non-bacterial hazards are also acknowledged where they are relevant to disease burden, representative detection platforms, and food-safety surveillance.

Current foodborne pathogen detection involves several methodological routes, including culture-based methods, immunological assays, nucleic-acid-based techniques, biosensing platforms, and emerging integrated analytical approaches. Culture-based methods are still used as reference methods because they provide reliable results, recover viable isolates, and support regulatory confirmation. However, most culture-based workflows require long assay times, intensive manual operation, and multiple confirmation steps. They also show limited compatibility with viable but non-culturable (VBNC) pathogens. These features restrict their use in rapid screening, high-throughput testing, and emergency response [1,7]. Other methods address part of these limitations. Immunological assays, nucleic acid amplification and recognition methods, and biosensors have improved the speed, sensitivity, and flexibility of pathogen detection. Microfluidics, clustered regularly interspaced short palindromic repeats (CRISPR)-Cas systems, mass spectrometry, sequencing technologies, and artificial intelligence (AI)-assisted analysis are also being integrated with portable devices and data-analysis tools. These combinations support more compact, field-oriented, and multiplex detection platforms. However, practical performance still depends on sample pretreatment, matrix compatibility, and workflow stability.

Recent research has moved beyond the improvement of single-assay sensitivity or speed. More attention is now given to the complete analytical workflow. This workflow includes sample pretreatment, target enrichment, signal generation, result readout, and data interpretation. Immunomagnetic separation (IMS) can improve target-cell recovery from complex food matrices. Isothermal amplification and CRISPR-Cas systems can provide rapid nucleic acid recognition with relatively simple equipment. Microfluidic chips can integrate sample handling, reaction, and detection in sample-in-result-out formats. AI and big-data analytics can support signal interpretation, contamination trend analysis, and risk prediction [8]. These advances have not removed the main barriers in real food testing. Food-matrix components may interfere with nucleic acid extraction, amplification, target recognition, or signal readout. Many methods also cannot clearly distinguish viable cells from dead-cell-derived nucleic acids. Validation also remains insufficient, especially for naturally contaminated foods. For field-deployable systems, device stability, cross-platform comparability, and standardization remain important requirements.

Several reviews have summarized foodborne pathogen detection methods, but many of them have focused on conventional assays or on a single technical route. Fewer reviews have compared culture-based methods, immunological assays, nucleic acid technologies, biosensors, microfluidics, mass spectrometry, sequencing, and artificial intelligence-assisted analysis within the same practical framework. As a result, the connections among regulatory confirmation, rapid screening, point-of-need testing, viable-cell discrimination, sample pretreatment, and data interpretation remain insufficiently discussed.

**Figure 1:**
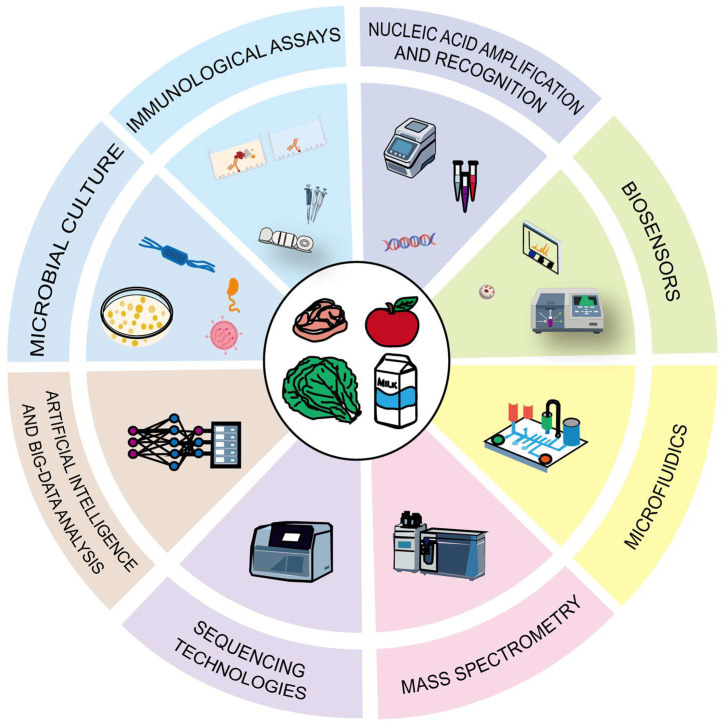
Schematic overview of representative analytical strategies for foodborne pathogen detection in food matrices.

This review addresses this gap by evaluating both established and emerging technologies from an application-oriented perspective. The comparison focuses on analytical principles, performance, sample pretreatment, viable-cell discrimination, matrix compatibility, field applicability, and translational potential. Based on this framework, this review further highlights several emerging trends, including integrated sample-to-result workflows, closed and portable detection systems, matrix-compatible pretreatment, viable-cell-relevant analysis, multiplex detection, and AI-assisted signal interpretation (Figure 1).

The literature for this narrative review was mainly collected from Web of Science, Scopus, and PubMed. Recent reviews, methodological studies, and representative applications published in recent years were prioritized. Classical papers were included when they were necessary to explain key detection principles.

In addition to qualitative comparison, quantitative performance metrics are necessary for evaluating the practical value of different detection methods. Parameters such as limit of detection, assay time, throughput, cost, sample pretreatment requirement, and validation sample type directly determine whether a method can be used for regulatory confirmation, rapid screening, or point-of-need testing. However, these metrics are often reported under different experimental conditions and are not always directly comparable. Therefore, this review compares representative performance ranges rather than fixed universal values.

Validation level is another important factor for evaluating practical applicability. Many rapid detection platforms are first tested using pure cultures, buffer systems, or artificially spiked food samples. These settings are useful for controlled method development, but they do not fully reproduce real contamination scenarios. In naturally contaminated foods, pathogens may occur at low levels, show uneven distribution, and coexist with complex background microbiota. They may also be stressed, injured, attached to food particles, or present in a VBNC state. Therefore, performance obtained from artificially spiked samples should not be directly interpreted as routine performance in real food testing.

Table 1 summarizes representative foodborne pathogen detection methods using both qualitative and quantitative criteria. These criteria include analytical principle, major strengths, limitations, typical detection level, assay time, throughput, cost, application scenarios, and validation level. Because reported performance values vary across pathogen species, food matrices, pretreatment procedures, enrichment strategies, and readout formats, the table presents representative ranges or qualitative estimates rather than fixed universal values. Through this framework, this review aims to clarify the roles of different methods in regulatory confirmation, rapid screening, point-of-need testing, and future integrated food safety monitoring.

## 2. Conventional Detection Methods

### 2.1. Culture-Based Isolation and Biochemical Identification

Culture-based isolation followed by biochemical identification remains a reference approach for foodborne pathogen detection. It is still required in many regulatory workflows because it enables viable-cell recovery, isolate confirmation, downstream typing, and antimicrobial resistance analysis [16]. Although this method requires a long assay time and has limited ability to detect VBNC pathogens, it remains the benchmark for evaluating many rapid detection technologies. In routine food testing, culture enrichment is often the first step. This applies to different food matrices, including retail poultry, beef, dairy products, seafood, ready-to-eat foods, and fresh produce. Major foodborne bacterial pathogens, such as *Salmonella enterica* serovar Typhimurium (*S.* Typhimurium), pathogenic *Escherichia coli* (*E. coli*) including *E. coli* O157:H7, *Listeria monocytogenes* (*L. monocytogenes*), *Bacillus cereus* (*B. cereus*), *Staphylococcus aureus* (*S. aureus*), *Vibrio parahaemolyticus* (*V. parahaemolyticus*), *Vibrio cholerae* (*V. cholerae*), *Campylobacter jejuni* (*C. jejuni*), *Campylobacter coli* (*C. coli*), and *Yersinia enterocolitica* (*Y. enterocolitica*), are commonly detected through enrichment-based workflows. This step is especially important when serotyping, virulence characterization, or antimicrobial susceptibility testing is required.

A conventional plating workflow usually includes non-selective pre-enrichment, selective enrichment, isolation on selective or differential media, and biochemical or serological confirmation. The method uses physiological and metabolic differences among microorganisms, including nutrient requirements, growth conditions, and biochemical reactions. By adjusting medium composition, incubation temperature, pH, and atmospheric conditions, the target pathogen can be enriched while part of the background flora is suppressed. After incubation, suspected colonies are selected according to morphology, pigmentation, transparency, or hemolytic features. Further confirmation is performed by Gram staining, biochemical reactions, serological assays, or mass spectrometry [9]. Chromogenic media have improved the convenience of plate-based analysis. These media contain enzyme-specific substrates, allowing target pathogens to form colonies with characteristic colors. This supports preliminary identification and reduces errors caused by manual observation. Common examples include XLD, SS, and HE agar for *Salmonella* isolation, sorbitol MacConkey agar and related chromogenic media for *E. coli* O157:H7, Oxford agar for *L. monocytogenes*, and TCBS agar for *V. parahaemolyticus* [16]. Despite these improvements, enrichment, isolation, and identification remain core steps in many official detection methods.

Although the above workflow mainly describes bacterial culture-based detection, conventional detection of non-bacterial foodborne hazards follows different principles. Foodborne protozoa, such as *Cryptosporidium* spp., *Giardia duodenalis*, and *Cyclospora cayetanensis*, are not detected by routine bacterial culture workflows and usually require sample concentration, microscopy, staining, immunofluorescence assays, or molecular confirmation [17]. Foodborne fungi can be isolated on selective fungal media and identified by morphology, mass spectrometry, or molecular assays, whereas helminth parasites are more often detected by visual inspection, artificial digestion, microscopy, serological assays, or molecular methods [18]. Therefore, non-bacterial foodborne hazards require distinct sample preparation and confirmation strategies, although this review mainly focuses on bacterial culture-based detection.

### 2.2. Integration of Culture-Based Methods with Rapid Confirmation Tools

Culture-based methods are still time-consuming. Their efficiency can be improved by introducing rapid identification tools after enrichment and isolation. One common strategy is to combine culture enrichment with matrix-assisted laser desorption/ionization time-of-flight mass spectrometry (MALDI-TOF MS). Once a pure colony is obtained, MALDI-TOF MS identifies the isolate by analyzing its protein fingerprint. This approach can shorten the confirmation stage [13]. As of now, this combined workflow has been used to identify *S.* Typhimurium in retail chicken and beef. It has also been applied to the detection and identification of *E. coli*, *S.* Typhimurium, *Klebsiella*, and *Enterobacter* in fish, meat, and dairy products [19]. In fresh vegetable samples, MALDI-TOF MS has been used for rapid confirmation of *E. coli*, *S.* Typhimurium, *S. aureus*, *C. jejuni*, and *L. monocytogenes* [20]. These studies indicate that culture-based workflows are not being replaced directly. Instead, they are increasingly supplemented with instrumental confirmation after enrichment and isolation.

Overall, culture-based methods remain central to regulatory testing. They are low-cost, standardized, easy to interpret, and able to recover viable isolates. Nevertheless, their limitations are also clear. Most workflows require 2–7 days and involve several labor-intensive steps. VBNC pathogens may be missed because they do not readily form visible colonies under routine culture conditions. Target recovery and identification can also be affected by complex food matrices and competitive background flora [21]. Certainly, culture-based methods are unlikely to be fully replaced in the near term. A more practical direction is to use them as reliable isolation or confirmation steps, while integrating immunological assays, nucleic acid methods, and biosensing platforms to improve the speed and efficiency of the overall work.

## 3. Immunological Methods

### 3.1. Enzyme-Linked Immunosorbent Assay (ELISA)

Immunological methods are mainly based on specific antigen–antibody recognition [1]. They are widely used for rapid screening of foodborne pathogens because they are relatively simple, fast, and suitable for batch analysis. Compared with plate-based culture methods, which often require several days, many immunological assays can produce results within 1–6 h and require less complex instrumentation [9]. These methods are not always sufficient for final confirmation, but they are useful for front-end screening and preliminary risk assessment.

**Figure 2:**
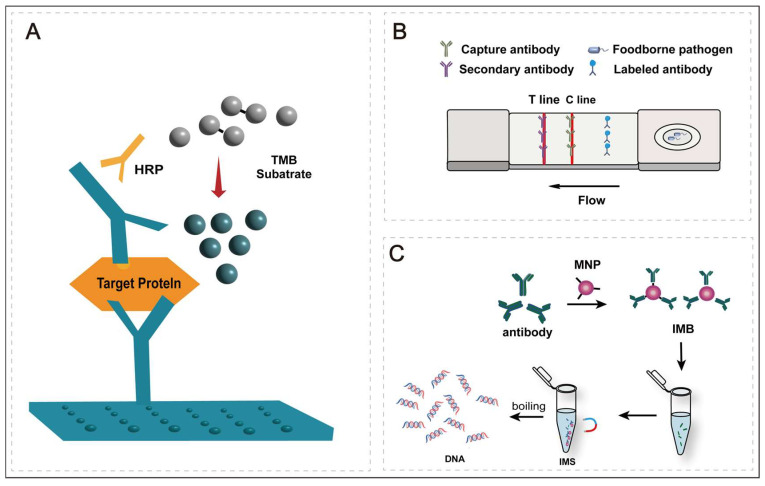
(**A**) Schematic diagram of the ELISA principle. (**B**) Schematic diagram of the LFIA principle. (**C**) Schematic diagram of the IMS method.

ELISA is one of the most established immunological methods. In a typical ELISA format, capture antibodies are immobilized on a microplate to bind the target antigen (Figure 2A). Enzyme-labeled secondary antibodies then react with a substrate to generate a visible or instrument-readable signal. The signal intensity is usually related to the target concentration. Depending on the assay design, ELISA can be performed in sandwich, indirect, or competitive formats. It has been used for the detection of *Salmonella*, *B. cereus*, *L. monocytogenes*, *E. coli* O157:H7, and *V. parahaemolyticus* [8].

Several modified ELISA formats have been reported to improve sensitivity, simplify operation, and enhance compatibility with food samples. These formats mainly involve new assay carriers, signal amplification, and integration with nucleic acid amplification or target-enrichment steps. Paper-based ELISA has also been explored for *E. coli* O157:H7 detection [22]. In addition, PCR-ELISA can combine nucleic acid amplification with immunological signal readout. For example, duplex PCR-ELISA has been developed for simultaneous detection of *Salmonella* spp. and *E. coli* O157:H7 in food samples, showing improved sensitivity and specificity compared with conventional PCR-based readout [23].

Despite these improvements, ELISA is still mainly suited to laboratory-based screening and batch analysis. Low-abundance targets often require pre-enrichment, and the assay is not usually sufficient for independent confirmatory testing. Its practical value therefore lies in rapid screening rather than final confirmation.

### 3.2. Immunochromatographic Assays (ICA)

ICA, especially lateral flow immunoassays (LFIAs), are commonly used for rapid on-site screening. LFIA usually uses a nitrocellulose membrane as the carrier. The sample moves along the strip by capillary flow, and antigen–antibody recognition occurs at the test line. A visible result can often be obtained within 5–15 min (Figure 2B). Because LFIA is portable, low-cost, and easy to operate, it is suitable for field screening and resource-limited settings [24].

Traditional colloidal-gold LFIA uses gold nanoparticles as colorimetric labels. The result can be read without additional instruments. More recent LFIA systems use fluorescent microspheres, magnetic particles, quantum dots, or other signal labels to improve sensitivity and semi-quantitative performance. These modifications have allowed LFIA to move from simple qualitative testing toward multiplex and more quantitative analysis.

LFIA has been used for the rapid detection of *E. coli* O157:H7, *Salmonella*, and other foodborne pathogens in food samples [25]. Machine-learning-assisted dual-channel catalytic immunochromatographic systems have been reported to improve analytical sensitivity [26]. Nanobody-functionalized magnetic–fluorescent nanoprobes have also been used to construct dual-signal LFIA systems for multiplex foodborne pathogen detection [27].

Even so, conventional LFIA still has limited sensitivity and quantitative precision for low-abundance targets. Its result may also be influenced by label stability and visual-readout subjectivity. Therefore, LFIA is mainly suitable for rapid preliminary screening, while broader quantitative use requires improved signal amplification and more standardized readout strategies [27].

### 3.3. Immunomagnetic Separation

IMS is mainly used as a pretreatment and enrichment strategy rather than as an independent endpoint assay. Its core material is superparamagnetic beads coated with specific antibodies (Figure 2C). After binding to the target pathogen, the bead–bacteria complexes can be collected by an external magnetic field. This process helps concentrate low-abundance targets and reduce background interference [28].

In foodborne pathogen detection, IMS is often coupled with ELISA, PCR, LFIA, real-time LAMP, or MALDI-TOF MS. Common integrated workflows include IMS-ELISA, IMS-PCR, and IMS-LFIA. These combinations are useful for detecting low-abundance *Salmonella*, *L. monocytogenes*, and *Campylobacter* in complex food matrices. For example, immunomagnetic beads combined with real-time LAMP have been used for rapid and sensitive detection of *Salmonella* in ready-to-eat duck meat [28]. Nanobody-based IMS platforms have also been developed for the rapid separation and detection of *Salmonella enteritidis* (*S. enteritidis*) in food samples [29].

IMS improves target recovery before downstream analysis and functions as an upstream enrichment module in rapid detection workflows. Its performance depends on bead quality, antibody specificity, binding efficiency, and non-specific adsorption. For routine use, more attention should be given to bead reproducibility and compatibility with automated detection platforms.

Conclusively, immunological methods remain useful for rapid foodborne pathogen detection because they balance speed, portability, cost, and operational simplicity. Advances in nanomaterials, engineered antibodies, nanobodies, microfluidics, and intelligent readout have improved their sensitivity, multiplexing capacity, and automation [30]. In practical food testing, however, these methods are better positioned for screening or enrichment-assisted detection. Their routine application still depends on affinity-reagent quality, antigen accessibility, matrix compatibility, and validation using naturally contaminated foods. Final confirmation usually requires culture-based or molecular methods.

## 4. Nucleic Acid Amplification and Recognition Methods

Nucleic acid amplification and recognition methods are widely used for foodborne pathogen detection because they target pathogen-specific genetic sequences. Compared with culture-based and immunological methods, they usually provide higher sensitivity and specificity, especially for low-abundance contamination. Common readout formats include fluorescence, colorimetry, electrophoresis, and lateral-flow detection. PCR and its variants, isothermal amplification, DNA microarrays, and CRISPR-Cas systems now form the main molecular toolbox for rapid laboratory testing and integrated detection platforms [31]. Their performance in food testing, however, still depends on sample pretreatment, matrix inhibition, viable/dead-cell discrimination, and instrument requirements.

### 4.1. Polymerase Chain Reaction (PCR) and Its Variants

**Figure 3:**
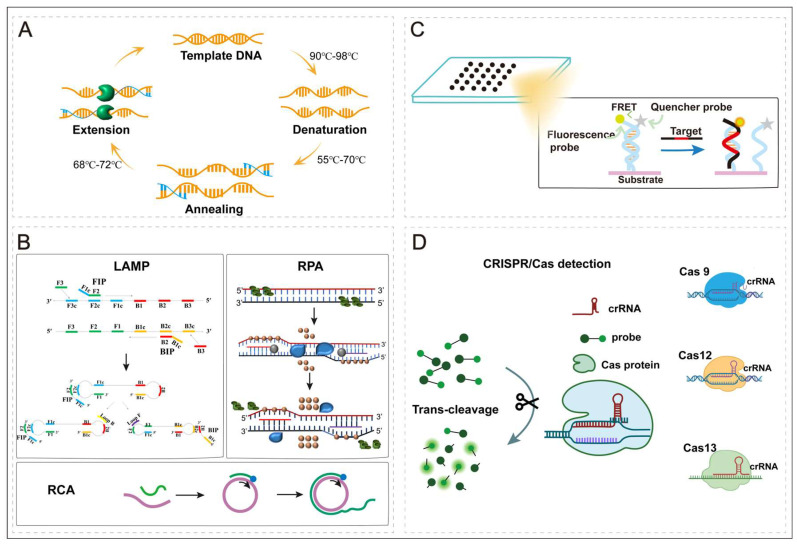
(**A**) Schematic diagram of the PCR principle. (**B**) Schematic diagram of representative isothermal amplification methods. (**C**) Schematic diagram of DNA microarrays. (**D**) Schematic diagram illustrating the principles of the CRISPR-based diagnostic system.

PCR is a mature molecular method for detecting foodborne pathogens (Figure 3A). It amplifies target DNA fragments through repeated denaturation, annealing, and extension cycles. The products can then be analyzed by gel electrophoresis, fluorescence monitoring, or endpoint counting [32]. PCR-based methods have been used for *L. monocytogenes*, *E. coli* O157:H7, *S. aureus*, *C. jejuni*, *Salmonella*, and *Shigella*. They have also become part of several standard detection workflows. Compared with culture-based methods, PCR shortens assay time. It still requires thermal cycling equipment, controlled laboratory conditions, and contamination control. Matrix inhibition and signals from dead-cell-derived DNA can also affect the interpretation of the results [10].

Multiplex PCR (mPCR) is used when several pathogens need to be screened in one reaction. It introduces multiple primer pairs into the same system, allowing different target genes to be amplified in parallel [33]. This design reduces assay time and reagent use. It also improves screening throughput. In ready-to-eat foods and other complex matrices, mPCR has been used to detect *E. coli*, *L. monocytogenes*, *S. aureus*, and *Salmonella* in the same assay [34]. The method still requires careful primer design. Different targets may not amplify with the same efficiency. Therefore, sensitivity can vary with the target combination, primer system, and food matrix.

Quantitative PCR (qPCR) adds real-time fluorescence monitoring to conventional PCR. Fluorescent dyes or probes record amplification signals, and the target concentration is estimated from Ct values and standard curves. This improves quantitative capacity, repeatability, and contamination control [8]. qPCR has been applied to *Salmonella*, *L. monocytogenes*, *E. coli* O157:H7, and aquatic-product-related pathogens in dairy, meat, fruit, vegetable, and seafood samples [35].

Digital PCR (dPCR) uses a different quantification strategy. It partitions the reaction mixture into many micro-reactions and calculates the target copy number from the number of positive partitions. This enables absolute quantification without standard curves. dPCR is often more tolerant of matrix interference and can be useful for low-copy targets, but its routine use is limited by instrument cost and workflow complexity [36,37]. Even so, dPCR is not yet easy to apply on a large scale because the instruments are costly and the workflow is relatively complex.

Viability PCR (vPCR) was developed to reduce the influence of dead-cell-derived DNA. It uses membrane-impermeable dyes such as propidium monoazide (PMA) or ethidium monoazide (EMA) to suppress amplification of DNA from membrane-damaged or dead cells [38]. In this way, the detected signal is more closely related to viable-cell contamination. vPCR has been used in ready-to-eat foods, drinking water, and aquatic products. It has also been applied to disinfection evaluation, shelf-life monitoring, and microbial risk assessment [39]. Nevertheless, vPCR performance is strongly influenced by dye concentration, light exposure conditions, and sample-matrix properties.

In addition to vPCR, PMA/EMA-qPCR is an important strategy for improving viable-cell relevance in quantitative nucleic acid detection [40]. RNA-based detection provides another option. Because mRNA is usually less stable than DNA and is more closely associated with active gene expression, RT-qPCR or isothermal amplification targeting mRNA can make the detection signal more relevant to metabolically active cells [41]. rRNA targets may also be useful because they are abundant in bacterial cells, although their persistence after cell death should be considered. These strategies improve viable-cell relevance, but they are still affected by dye concentration, light exposure, food-matrix turbidity, RNA stability, and bacterial stress states.

Representative studies further clarify the different roles of PCR-based variants in foodborne pathogen detection. For multiplex screening, Boukharouba et al. developed an mPCR assay targeting *E. coli*, *L. monocytogenes*, *S. aureus*, and *Salmonella* in organic lettuce and minced meat, using *gadA*, *LM404*, *nuc*, and *invA* as target genes, with a reported detection limit of 10^2^ CFU/mL and an assay time of approximately 30 h [34]. This study supports the use of mPCR for simultaneous screening of multiple pathogens in complex food matrices. At the same time, it also indicates that enrichment steps and primer-balance optimization are still important for maintaining sensitivity and assay consistency. For viable-cell-relevant analysis, Liu et al. reported a PMA-assisted mPCR assay for the simultaneous detection of viable *E. coli* O157:H7, *S. aureus*, and *Salmonella* in milk and ground beef. The assay targeted *rfbE*, *nuc*, and *invA*, respectively, and achieved a detection limit of 10^4^ CFU/mL within 4–6 h [42]. The PMA-assisted mPCR example indicates that viability dyes can make PCR results more closely related to viable-cell contamination by suppressing signals from dead-cell-derived DNA. dPCR serves a different purpose. Rather than improving viable/dead-cell discrimination, it provides absolute quantification of low-copy targets without the use of standard curves. For example, Wang et al. used dPCR to detect *S.* Typhimurium in milk by targeting *FimY*, achieving a detection limit of 10^2^ CFU/mL [37]. Taken together, conventional PCR and qPCR are mainly used for confirmation and quantification. mPCR is suitable for multi-pathogen screening. PMA/EMA-assisted PCR improves viable-cell relevance. dPCR provides high-precision copy-number analysis. Broader use still depends on efficient nucleic acid extraction, reduced matrix inhibition, strict contamination control, and validation in naturally contaminated samples.

### 4.2. Isothermal Amplification Methods

Isothermal amplification is useful when detection needs to be performed outside well-equipped laboratories. Unlike PCR, it amplifies nucleic acids at a constant temperature and does not require thermal cycling equipment. This makes the method easier to combine with visual readouts, portable readers, and microfluidic devices. Common strategies include loop-mediated isothermal amplification (LAMP), rolling circle amplification (RCA), and recombinase polymerase amplification (RPA) (Figure 3B). In food analysis, LAMP and RPA are used more often because they are easier to integrate into rapid workflows.

LAMP uses multiple primers to recognize several regions of the target sequence. With a strand-displacing polymerase, it can rapidly amplify nucleic acids under isothermal conditions [43]. Its readout can be based on turbidity, fluorescence, colorimetry, or lateral-flow formats. LAMP has been used for *E. coli*, *Salmonella*, *V. cholerae*, and *L. monocytogenes* [44]. It can also be integrated with microfluidic chips for multiplex detection. The main limitations are complex primer design and non-specific amplification.

RCA is more often used as a signal amplification strategy than as a stand-alone pathogen detection method. It uses circular DNA as the template and continuously generates long single-stranded DNA molecules with repeated sequences [45]. This process amplifies the signal and can be coupled with nucleic acid probes, microfluidic chips, and fluorescence systems [46]. RCA has been used for *E. coli* O157:H7 in milk, juice, and drinking water, and for visual detection of *S. aureus* and *S. enterica* [47,48,49].

RPA is suitable for field-oriented testing because it works at a relatively low temperature and produces detectable products within a short time. Under typical conditions, RPA operates at approximately 37–42 °C and generates signals within 10–20 min [50]. It can be combined with lateral-flow strips, handheld fluorescence readers, and other portable devices [51,52]. RPA–lateral flow dipstick (RPA-LFD) has been used to detect *E. coli* O157:H7, *Salmonella*, and *Shigella* in raw meat [53]. Related studies have also reported visual detection of *Salmonella invA* in mutton, chicken, and broccoli samples [51]. Routine use of RPA is still limited by reagent cost, intellectual-property restrictions, and non-specific amplification under suboptimal conditions.

The practical value of isothermal amplification lies in its simple temperature requirement, short assay time, and compatibility with portable readouts. LAMP mainly supports integrated and multiplex analysis. RPA is more suitable for rapid field screening. RCA is often used in signal-amplification systems. Reported results, however, are not always directly comparable because detection limits, assay times, and sample types differ across studies. These differences are often linked to sample pretreatment, enrichment procedures, and readout formats. Future work should focus on closed-tube operation, reduced non-specific amplification, reagent stability, standardized evaluation, and validation with real food samples.

Representative examples illustrate these differences. These studies differ in amplification strategy, target pathogens, food matrices, readout format, detection time, and reported detection limits. Cao et al. developed a microfluidic chip-based LAMP platform for simultaneous detection of *Salmonella*, *S. aureus*, *E. coli* O157:H7, and *Shigella* in aquatic-product-related samples, with a detection limit of 8 × 10^3^ CFU/mL within 60 min [54]. Bai et al. developed an RPA-LFD assay for *E. coli* O157:H7, *Salmonella*, and *Shigella* in raw meat, reaching 10 CFU/mL within 35 min [53]. Yang et al. used saltatory RCA for visual detection of *S. aureus* in milk, with a detection limit above 5.6 × 10^2^ CFU/mL within 60 min [47]. These examples support the use of isothermal amplification in rapid and portable detection, but broader application still requires improved primer/probe design and stronger evidence from naturally contaminated foods.

### 4.3. DNA Microarray Technology

DNA microarrays enable parallel analysis of multiple nucleic-acid targets. Known oligonucleotide probes are immobilized on a solid surface and hybridize with complementary target sequences (Figure 3C). Compared with single-target amplification methods, DNA microarrays are better suited to information-rich laboratory analysis. They can be used for pathogen identification, virulence gene screening, antimicrobial-resistance gene analysis, and molecular typing [55].

Reported applications show their value in high-throughput analysis. Direct 16S rRNA hybridization-based microarrays have been developed for multiplex pathogen detection by using conserved and species-specific ribosomal RNA sequences [56]. Signal-probe-based DNA microarrays have also been explored for amplification-free or near-amplification-free detection of bacterial genes. One platform detected bacterial 5S rRNA within about 30 min [57]. Simplified microarray formats that reduce labeling and washing steps have also been reported [58].

Even so, DNA microarrays remain mainly laboratory-based. Conventional workflows involve nucleic acid extraction, labeling, hybridization, washing, imaging, and data interpretation. These steps increase assay time, operational complexity, and instrument requirements. Therefore, DNA microarrays are more suitable for comprehensive genetic analysis than rapid point-of-need testing. Broader use in food testing will require simplified workflows, lower-cost platforms, and automated data analysis.

### 4.4. CRISPR-Cas System-Based Detection

CRISPR-Cas systems have become important tools in nucleic-acid-based foodborne pathogen detection. Their main advantage is the additional sequence specificity provided by programmable guide RNAs. Cas9, Cas12, and Cas13 can be directed to specific nucleic acid targets (Figure 3D). Some Cas systems also show collateral cleavage activity, which can cleave reporter probes and generate fluorescence, colorimetric, or lateral-flow signals [59,60]. These features make CRISPR-Cas systems suitable for rapid detection when combined with portable readout formats.

A typical CRISPR-Cas workflow includes nucleic acid release, pre-amplification, target recognition, and signal readout. The method can be adapted to different pathogens, food matrices, pre-amplification strategies, and readout systems. Gong et al. developed a one-pot RPA-CRISPR/Cas12b assay for *Salmonella enterica* serovar Indiana in chicken carcass samples. The assay reached 14.4 copies/reaction within less than 60 min [61]. Gao et al. reported a PCR-coupled LwCas13a platform for *Salmonella* detection in milk and juice, with a detection limit of 1 CFU/mL within 120 min [62].

CRISPR-Cas systems can also be designed for multiplex detection. Xing et al. combined Cas12a with recombinase-aided amplification for simultaneous detection of *S. aureus*, *B. cereus*, *P. aeruginosa*, *Cronobacter sakazakii*, *L. monocytogenes*, *V. parahaemolyticus*, and *S.* Typhimurium in pasteurized milk and purified drinking water. The assay achieved 5 × 10^2^ CFU/mL within 62 min [63]. Fang et al. developed an RPA-CRISPR/Cas12a-coupled microfluidic biosensor for on-site quantification of *V. parahaemolyticus* in seafood. The reported detection limit was 6.08 copies/μL, with an assay time of 90 min [64]. These studies show that CRISPR-Cas platforms are moving from proof-of-concept nucleic acid recognition toward faster, multiplex, and more integrated detection formats [65]. In particular, one-pot droplet microfluidic CRISPR/Cas13a systems have recently been developed for multiplex bacterial detection, suggesting that CRISPR-Cas platforms are increasingly moving toward compartmentalized and high-throughput formats [66,67].

Routine use of CRISPR-Cas detection in food analysis is still constrained by workflow-level issues [68]. Complex food matrices may interfere with nucleic acid release, purification, pre-amplification, and signal readout. Most CRISPR-Cas assays detect nucleic acid targets rather than viable cells directly. Therefore, dead-cell-derived DNA or RNA may contribute to the final signal if no viability-linked pretreatment or target-selection strategy is included. Viable-cell relevance can be improved by combining CRISPR-Cas detection with culture enrichment, PMA/EMA pretreatment, RNA-targeted detection, or phage-assisted strategies [41]. PMA/EMA pretreatment can be introduced before nucleic acid amplification to reduce signals from dead-cell-derived DNA [39]. RNA-targeted CRISPR detection, especially assays targeting mRNA or other short-lived transcripts, may better reflect metabolically active cells than DNA-targeted assays [69]. Phage-assisted strategies are another possible route because phage replication or phage-induced nucleic acid signals are more closely associated with viable host cells [70]. However, these approaches also introduce additional workflow requirements. RNA is vulnerable to degradation, PMA/EMA treatment is affected by sample turbidity and cell membrane integrity, and phage-based methods depend on host range and infection efficiency. In addition, many CRISPR-Cas platforms still require a separate pre-amplification step, which increases workflow complexity and may raise the risk of carryover contamination if the system is not closed. Future development should therefore focus on matrix-compatible sample preparation, closed-tube or integrated operation, viable-cell-relevant analysis, and validation with naturally contaminated food samples.

## 5. Biosensor-Based Detection Methods

Biosensors couple a biological recognition element with a physicochemical transducer. The recognition element captures or identifies the target pathogen. The transducer then converts this event into an electrical, optical, or mass-sensitive signal. This format is suitable for rapid foodborne pathogen detection because it supports fast response, miniaturization, and portable readout. Recent biosensor platforms often combine recognition elements with nanomaterials, nucleic acid amplification, microfluidics, and intelligent signal analysis to improve sensitivity, integration, and field applicability [71,72].

### 5.1. Electrochemical Biosensors

**Figure 4:**
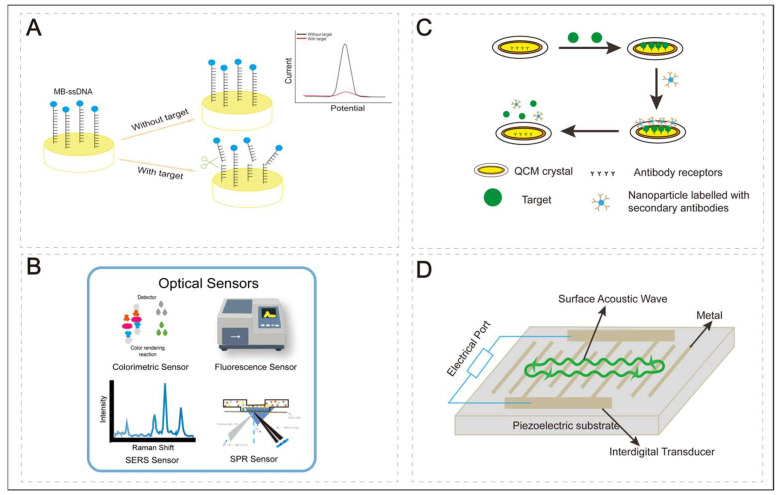
(**A**) Schematic diagram of a typical electrochemical biosensor. (**B**) Schematic diagram of a typical optical biosensor. (**C**) Schematic diagram of the detection principle of the quartz crystal microbalance (QCM)-based immunosensor. (**D**) Schematic diagram of the basic structure of a surface acoustic wave (SAW) biosensor.

Electrochemical biosensors are among the most widely used biosensor formats for foodborne pathogen detection. Their principle is to convert the interaction between a pathogen and a recognition element into a measurable electrochemical response. The signal may appear as a change in current, potential, conductivity, impedance, or capacitance [73]. Compared with many optical or mass-sensitive systems, electrochemical platforms usually require simpler instruments. They also respond quickly and are compatible with portable devices. These features make them suitable for field-oriented analysis (Figure 4A).

Recognition elements determine the analytical function of electrochemical biosensors. Immunosensors use antigen–antibody recognition and are useful for rapid screening in complex food samples. Aptasensors use nucleic-acid aptamers and offer advantages in stability, chemical modification, and cost. Phage-based sensors provide host-specific recognition and may support viable-cell-associated detection. Nucleic-acid-based electrochemical platforms, especially those combined with CRISPR/Cas12a or Cas14a, can improve sequence-recognition specificity and multiplexing capacity [74,75,76].

Electrode modification is widely used to improve signal transfer and recognition efficiency. Graphene, carbon nanotubes, MXenes, metal–organic frameworks, metal nanoparticles, and core–shell composites can increase the active surface area, enhance electron transfer, and improve the loading of recognition molecules. Some systems have achieved detection limits as low as 10^0^–10^2^ CFU/mL. Electrochemical biosensors have been used for *E. coli* O157:H7, *Salmonella*, *Listeria*, *S. aureus*, and *V. parahaemolyticus* in dairy, meat, aquatic products, fruits, and vegetables. Reported examples include gold-nanoparticle-modified screen-printed carbon electrodes for *E. coli* O157:H7 and metal–organic-framework-assisted platforms for simultaneous multi-pathogen detection. Despite these advances, routine use still depends on sensor stability, batch-to-batch reproducibility, anti-fouling performance, and matrix compatibility.

### 5.2. Optical Biosensors

Optical biosensors detect pathogens through changes in optical signals. Common signal types include fluorescence, color variation, Raman scattering, refractive-index changes, and resonance shifts (Figure 4B). Some optical platforms allow direct or real-time analysis. Others can be read visually or with simple optical devices, which is useful for rapid screening in settings with limited instrumentation [77]. For foodborne pathogen detection, optical biosensors mainly include fluorescence sensors, colorimetric sensors, surface-enhanced Raman scattering (SERS) sensors, and surface plasmon resonance/localized surface plasmon resonance (SPR/LSPR) sensors [78].

#### 5.2.1. Fluorescence Sensors

Fluorescence sensors usually rely on fluorescent dyes, quantum dots, upconversion nanoparticles, carbon dots, or aggregation-induced-emission materials. After target binding, detection is achieved by monitoring fluorescence intensity, wavelength shifts, or energy-transfer changes [78]. Fluorescence-based assays are often more sensitive than colorimetric methods and are compatible with multiplex and quantitative readouts. They have been used for *E. coli* O157:H7, *Salmonella*, *S. aureus*, *L. monocytogenes*, and *V. parahaemolyticus*. Many systems also show compatibility with microfluidic chips and smartphone-based platforms [79]. In real food samples, however, background fluorescence, signal instability, and matrix-derived quenching can affect accuracy.

#### 5.2.2. Colorimetric Sensors

Colorimetric sensors convert target recognition into a visible color change. The result can be read by the naked eye or quantified with simple optical devices. Their signal generation is usually based on nanoparticle aggregation or disaggregation, enzyme-mediated chromogenic reactions, or catalytic color development using nanomaterials or enzyme-mimicking materials [80,81,82,83]. Colorimetric sensors are useful for preliminary screening because they require little instrumentation and provide intuitive outputs. AI-assisted smartphone colorimetric biosensors and 96-well immunocolorimetric platforms have been used for *E. coli* O157:H7, *Salmonella*, and *L. monocytogenes* in vegetables, meats, and dairy-related matrices. Their main limitations are robustness and quantitative reproducibility in highly complex food backgrounds.

#### 5.2.3. SERS Sensors

SERS sensors detect pathogens by enhancing Raman fingerprint signals. The enhancement is generated mainly by localized electromagnetic fields on nanostructured metal surfaces [84]. Since bacterial species and strains differ in cell-wall structure, membrane composition, and surface biomolecules, SERS can provide molecular fingerprints for fine-level differentiation. To reduce matrix interference, SERS is often combined with magnetic separation or enrichment [85,86]. Reported platforms based on AuNPs, structured silicon, or Ag-based nanostructures have been used for *E. coli* O157:H7, *S. enteritidis*, *P. aeruginosa*, *S. aureus*, and *B. cereus* in milk, water, meat, vegetables, and eggs. The main challenge is the reproducibility of signal enhancement, especially across different substrates and batches.

#### 5.2.4. SPR/LSPR Sensors

SPR and LSPR sensors enable label-free and real-time detection by monitoring refractive-index changes at the sensing interface. When target pathogens bind to antibodies, aptamers, phages, or other immobilized recognition molecules, the local refractive index changes and induces a shift in resonance angle or wavelength [87]. These systems are useful for interaction monitoring and kinetic analysis. They have been applied to *S. aureus*, *E. coli*, and *Salmonella*, and can be integrated with microfluidics to reduce sample consumption and improve automation [88,89]. Their broader use is still limited by cost, matrix effects, and the need for stable and reproducible surface functionalization.

### 5.3. Mass-Sensitive Biosensors

Mass-sensitive biosensors detect target binding by measuring interfacial mass changes. Based on the piezoelectric effect, these changes are converted into variations in frequency, phase, or wave velocity. The main platforms include quartz crystal microbalance (QCM) and surface acoustic wave (SAW) sensors. These systems can monitor binding events in a label-free or near-label-free manner and are useful for real-time analysis of microbial recognition [90].

#### 5.3.1. QCM Sensors

QCM uses a quartz crystal as the transducer. When recognition molecules immobilized on the electrode surface capture target pathogens, the surface mass increases while the resonance frequency decreases (Figure 4C). This enables real-time monitoring of bacterial binding [90]. Nanomaterial-enhanced interfaces, dissipation monitoring, and magnetic enrichment have been used to improve QCM performance for low-concentration targets. QCM has been applied to *E. coli* O157:H7 and *C. jejuni* in milk, burgers, dumplings, poultry rinse, and turkey mince [91,92].

#### 5.3.2. SAW Sensors

SAW sensors measure changes in acoustic waves propagating along piezoelectric surfaces (Figure 4D). These changes reflect interfacial mass, conductivity, and viscoelasticity. SAW systems usually have high operating frequency, good sensitivity, and integration potential. Flexible SAW immunosensors have been reported for *E. coli* detection. Love-mode SAW devices and thin-film SAW platforms have also been used for *S. aureus* and *E. coli* O157:H7 analysis [93,94]. For practical deployment, stability, anti-fouling performance, and matrix compatibility remain key issues.

Obviously, biosensors are useful for rapid, portable, and point-of-need foodborne pathogen detection. Their practical performance, however, is strongly affected by food-matrix compatibility and sensor reproducibility. Proteins, fats, pigments, salts, and background microorganisms in real food samples may interfere with target capture, recognition efficiency, and signal readout. Sensor stability, batch-to-batch consistency, storage conditions, and device calibration also affect routine use. Future studies should move beyond proof-of-concept sensitivity and include validation with naturally contaminated foods and inter-batch reproducibility testing.

Representative biosensor applications are summarized in Table 2. Although most reported biosensor studies still focus on bacterial foodborne pathogens, selected examples for non-bacterial foodborne hazards are also included to address the broader scope of foodborne pathogen detection. These examples include human norovirus, *Cryptosporidium parvum*, and *Trichinella spiralis*, representing foodborne viruses, protozoa, and helminth parasites, respectively.

## 6. Emerging Platforms for Integrated and Intelligent Foodborne Pathogen Detection

Recent work in foodborne pathogen detection has shifted from single-assay optimization to workflow integration and data-assisted analysis. These emerging platforms do not replace culture-based, immunological, or nucleic-acid-based methods directly. Instead, they extend the detection workflow at different stages. Microfluidic chips improve sample handling and process integration. Mass spectrometry supports rapid confirmation and microbial fingerprinting. Sequencing provides high-resolution genetic information. Artificial intelligence and big-data analysis improve signal interpretation, trend analysis, and risk-based decision support.

### 6.1. Microfluidic Chip Technology

**Figure 5:**
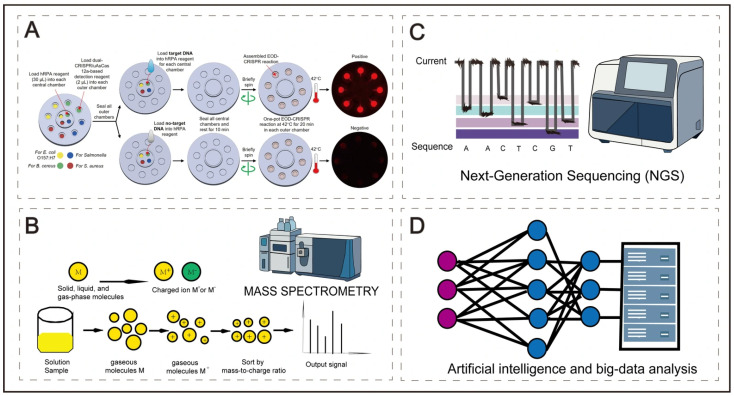
(**A**) Schematic diagram of the microfluidic detection principle [111]. Copyright 2025, WILEY. (**B**) Schematic diagram of the principles of mass spectrometry. (**C**) Schematic diagram illustrating the principles of next-generation sequencing (NGS). (**D**) Schematic diagram illustrating the detection principles based on artificial intelligence.

Microfluidic chip technology enables the controlled manipulation of nano- to picoliter-scale fluids in micrometer-scale channels (Figure 5A). Its main strengths are miniaturization, low sample consumption, and process integration. By integrating sample transport, separation, enrichment, reaction control, and signal detection on one chip, microfluidic systems can simplify multi-step analytical workflows. These features make them suitable for sample-in-result-out detection and point-of-care testing [12].

In foodborne pathogen detection, microfluidic chips are often used as integration platforms. They can be combined with optical, electrochemical, isothermal amplification, CRISPR-Cas, and SPR-based readouts. One useful design is the integration of microfluidics with magnetic-bead separation. Antibody- or aptamer-functionalized magnetic beads can capture target bacteria in microchannels. The bead–bacteria complexes are then separated by electromagnetic or hydrodynamic control. This process improves target enrichment and reduces non-specific interference from food matrices [112].

Centrifugal microfluidic chips have also been widely explored. In these systems, centrifugal force drives liquid movement, so external pumps are not required. This pump-free format is suitable for multi-channel operation and high-throughput analysis [113,114]. Microfluidics has also been combined with CRISPR-Cas systems and digital PCR to improve workflow control, automation, and analytical sensitivity. Some studies have reported detection limits close to 1 CFU/mL [115]. Recent studies further indicate that microfluidic platforms are moving toward closed, automated, and field-oriented sample-to-result systems. Fully enclosed microfluidic chip cartridges have been developed for point-of-care analysis of foodborne pathogens by integrating nucleic acid extraction, reagent preparation, LAMP reaction, and signal detection within a single device [112]. Gravity-driven microfluidic chips with tilt-actuated siphon valves have also been reported for rapid detection of *Salmonella* Typhimurium, reducing dependence on external pumps and complex fluid-control modules [116]. In addition, deep-learning-enhanced digital microfluidic chips have been used for multiplex detection of viable foodborne pathogens, showing the potential of combining microfluidic operation with intelligent image or signal interpretation [117]. Together, these studies suggest that microfluidic platforms are evolving from simple reaction miniaturization toward closed operation, simplified fluid control, multiplex viable-cell analysis, and intelligent readout.

Even so, microfluidics should be regarded as an enabling platform rather than a stand-alone detection method. Its value lies in combining sample handling, enrichment, reaction, and signal readout within a compact workflow. In real food testing, complex matrices may still cause channel blockage, poor mixing, reduced target recovery, or signal interference. Routine application also depends on chip fabrication, reagent storage, fluid-control modules, and reproducible large-scale production.

### 6.2. Mass Spectrometry

Mass spectrometry, especially MALDI-TOF MS, is used for rapid microbial identification. In food analysis, its main role is not primary screening of raw samples. It is more suitable for rapid confirmation after target organisms have been isolated or enriched (Figure 5B). MALDI-TOF MS identifies microorganisms by analyzing characteristic protein fingerprints [13,118].

MALDI-TOF MS has been applied to major foodborne pathogens, including *E. coli*, *Salmonella*, *L. monocytogenes*, and *Campylobacter*. It has also been incorporated into routine microbial identification workflows in some clinical and food microbiology laboratories [19,20]. The method is fast and does not require target-specific primers. However, its performance depends strongly on reference databases and spectral quality.

Mass spectrometry still has limitations in distinguishing closely related species. Complex sample backgrounds can also affect spectral interpretation [119,120]. Recent studies have therefore focused on improving spectral resolution, expanding reference databases, and applying more advanced data-analysis algorithms. For example, Dupré et al. developed a top-down-proteomics-based LC-MS/MS platform that improved the discrimination of phylogenetically close pathogens. This work shows that the future application of mass spectrometry will depend not only on instrument performance, but also on database quality and reliable spectral interpretation. In practical food testing, mass spectrometry is best positioned as a rapid confirmation and characterization tool after isolation or enrichment.

### 6.3. Sequencing Technologies

Sequencing technologies determine the nucleotide order of DNA or RNA and provide detailed genetic information. They are used for pathogen identification, typing, source tracing, and community analysis [121]. Compared with culture-based, immunological, and routine nucleic-acid-amplification methods, sequencing provides information beyond pathogen presence. It can reveal strain differences, virulence determinants, resistance genes, transmission relationships, and microbial community structure [14].

Different sequencing platforms have different roles. Sanger sequencing provides high accuracy and is useful for targeted fragment analysis, but its throughput is limited. Second-generation high-throughput sequencing enables large-scale parallel sequencing and is suitable for genomic surveillance and metagenomic analysis. Third-generation single-molecule sequencing, represented by nanopore sequencing, provides long reads, real-time analysis, and reduced GC bias [122].

In foodborne pathogen research, sequencing is used in outbreak investigation, molecular epidemiology, genomic surveillance, and metagenomic analysis of complex samples (Figure 5C). Next-generation sequencing (NGS) is especially useful for high-resolution source tracing. It can help identify cross-regional transmission and distinguish closely related isolates.

Routine use of sequencing in food testing is still limited by cost, sample pretreatment, bioinformatic requirements, and insufficient standardization. In complex food matrices, background microorganisms and host-derived nucleic acids may dilute pathogen-specific signals and complicate data interpretation. For this reason, sequencing is currently better suited to high-resolution confirmation, outbreak investigation, source tracing, and molecular characterization than to routine front-end screening of all food samples.

### 6.4. Artificial Intelligence and Big-Data Analysis

AI includes machine learning and deep learning methods that perform pattern recognition, classification, and prediction through data-driven training. Big-data analysis focuses on integrating large-scale and multi-source datasets to identify patterns and support risk-oriented decision-making. Unlike methods that directly capture, amplify, or sense pathogens, AI and big-data approaches mainly act on signals and datasets generated by other analytical platforms (Figure 5D). Therefore, their main value lies in result interpretation, data integration, automation, and decision support [15].

In foodborne pathogen detection, machine learning and deep learning models can be used for image-based result interpretation, smartphone colorimetric readout, automated colony counting, morphological classification, biosensor signal processing, spectral fingerprint analysis, sequencing data interpretation, and supply-chain monitoring. For example, convolutional neural networks are commonly used for image-based classification, while support vector machines, random forests, and partial least-squares models can assist spectral or sensor-signal classification. Recent studies have shown the value of these approaches. Deep learning has been integrated with digital microfluidic chips for multiplex detection of viable foodborne pathogens, and machine-learning-driven Raman spectroscopy has been used for rapid identification of foodborne pathogens with high classification performance. These models can help extract useful information from noisy backgrounds and reduce subjective manual judgment [117,123,124,125].

Big-data analysis is more relevant to food safety monitoring and early warning. Environmental monitoring data, supply-chain information, laboratory testing results, epidemiological data, and omics datasets can be integrated to build contamination-risk prediction models. These models can support real-time risk assessment, transmission-trend forecasting, and cross-stage tracing [126].

However, AI-assisted analysis does not eliminate the need for reliable sample preparation and stable analytical signals. Its performance depends on dataset size, data diversity, annotation quality, class balance, instrument consistency, and external validation. Annotated microbial image datasets are therefore important for developing and evaluating automated colony-counting and image-recognition models. Models trained on one food matrix, imaging condition, sensor batch, or laboratory dataset may not perform equally well under different conditions. Model interpretability, regulatory acceptance, and data-sharing standards also remain important barriers. Therefore, AI should be regarded as a complementary tool for signal interpretation, risk prediction, and decision support rather than as an independent replacement for validated detection methods [127].

## 7. Conclusions and Future Perspectives

Foodborne pathogen detection has moved beyond culture-based isolation and biochemical identification. It now involves a broader analytical system that includes immunological recognition, nucleic acid amplification, biosensing, microfluidics, mass spectrometry, sequencing, and artificial intelligence-assisted analysis. Each method has a distinct role. Culture-based methods remain essential for viable-cell isolation, regulatory confirmation, and downstream typing. Immunological assays, nucleic acid methods, and biosensing platforms are more suitable for rapid screening, multiplex analysis, and portable detection.

Practical application in real food samples remains difficult. Matrix interference can reduce target recovery, inhibit amplification, or affect signal readout. Low-abundance pathogens are also difficult to recover consistently. Many methods still cannot clearly distinguish viable cells from dead-cell-derived signals. In addition, validation is often based on artificially spiked samples, while data from naturally contaminated foods remain limited. Cross-platform compatibility is another unresolved issue. For many emerging technologies, analytical sensitivity is no longer the only criterion. Standardization, reproducibility, cost, field robustness, and reliable performance in complex food systems are equally important for routine use.

Future studies should give more attention to practical implementation. First, sample pretreatment should be more standardized and compatible with different food matrices. This is necessary for improving the recovery of low-abundance pathogens. Second, viable-cell-relevant detection should be strengthened through culture enrichment, PMA/EMA pretreatment, phage-based recognition, or RNA-targeted analysis. Third, validation should include more naturally contaminated foods rather than relying mainly on artificially spiked samples. Fourth, inter-laboratory comparison and standardized operating procedures are needed to improve reproducibility, regulatory acceptance, and industrial adoption.

Affordability and cost-effectiveness should also be considered when these technologies are translated into on-site applications. In low- and lower-middle-income countries, conflict-affected populations, and other resource-limited settings, the most suitable methods are not always those with the lowest detection limits or the highest level of automation. Practical implementation requires a balance among sensitivity, cost, reagent stability, ease of operation, maintenance burden, and infrastructure requirements. Culture-based methods, LFIA, simple immunoassays, and portable isothermal amplification platforms may remain useful for preliminary screening because they require relatively low equipment investment and can be adapted to decentralized testing. In contrast, highly integrated microfluidic systems, sequencing platforms, mass spectrometry, and AI-assisted analysis may provide stronger analytical capacity, but their broader use depends on instrument affordability, stable supply chains, electricity and internet access, trained personnel, and local validation.

Future research should not focus only on improving sensitivity. More effort is needed to integrate sample pretreatment, target enrichment, signal detection, and data interpretation into closed and user-friendly workflows. Multi-technology integration will be important, but it must be supported by robust devices, stable reagents, and standardized evaluation. Only when high analytical performance is combined with matrix compatibility, reproducibility, and field robustness can foodborne pathogen detection technologies move from proof-of-concept studies toward routine regulatory testing, industrial quality control, and practical food safety surveillance.

## Figures and Tables

**Table 1:** Overall comparison of foodborne pathogen detection methods.

Method Category	Target/Analyte	Basic Principle	Typical LOD	Assay Time	Throughput	Relative Cost	Validation Level	Major Strengths	Major Limitations	Application Scenarios & Examples
Conventional culture-based methods	Primarily culturable foodborne bacteria; extendable to some fungi	Isolation, purification, counting, and confirmation using selective and/or differential media	Usually qualitative after enrichment	24–72 h or longer	Low to medium	Low	Standardized	Direct and reliable results; relatively low cost; recovery of pure isolates; suitable for regulatory confirmation	Long assay time; labor-intensive; low throughput; poor ability to detect VBNC pathogens	Regulatory testing; isolate recovery; antimicrobial resistance analysis; epidemiological tracing
Immunological assays (ELISA, LFIA, IMS)	Mainly bacteria and toxins; extensible to fungi and viruses	Specific antigen–antibody recognition converts enzymatic chromogenic, fluorescent, colloidal-gold, or magnetic-bead signals into measurable outputs	10^3^–10^5^ CFU/g or CFU/mL	5 min–6 h	Medium to high	Low to medium	Mainly spiked	Rapid and convenient; relatively low cost; suitable for batch screening and front-end field screening; relatively low instrumentation dependence	Limited sensitivity; susceptible to matrix interference; usually unable to distinguish viable from dead cells	On-site food screening; batch sample analysis; target enrichment in pretreatment
Nucleic acid amplification and recognition technologies (PCR, LAMP, RPA, CRISPR-Cas)	Bacteria, fungi, viruses, and their specific nucleic acid sequences	Targeting pathogen-specific genes; signal amplification by thermal cycling or isothermal amplification with fluorescence, colorimetric, or lateral-flow readout	1–10^3^ CFU/g or CFU/mL	20 min–3 h	Medium to high	Medium	Spiked/standardized	High sensitivity and specificity; quantifiable; multiplex-capable; comparatively rapid	Susceptible to matrix inhibition; some methods require specialized equipment and trained personnel; limited viable/dead-cell discrimination	Rapid laboratory testing; quantitative analysis; low-abundance pathogen screening
Biosensors (electrochemical, optical, and mass-sensitive biosensors)	Bacteria, fungi, viruses, toxins, or marker molecules	Recognition elements such as antibodies, aptamers, nucleic-acid probes, or phages are coupled with transducers to convert binding events into electrical, optical, or mass-sensitive signals	Commonly 1–10^3^ CFU/mL or CFU/g	10–120 min	Low to medium	Medium	Mainly spiked	Fast response; high sensitivity; facile miniaturization and integration; strong potential for field use	Stability, batch consistency, and compatibility with complex matrices still need improvement	Point-of-need testing; online monitoring; portable analysis
Microfluidic platforms	Bacteria, viruses, fungi, toxins, and complex pretreatment systems	Integration of sample handling, separation, reaction, and detection in micro/nanoscale channels	Commonly 1–10^3^ CFU/mL or CFU/g after enrichment or integrated pretreatment	30–120 min	Medium	Medium to high	Mainly spiked	High integration and automation; low sample consumption; parallel analysis capability	Complex chip fabrication; demanding requirements for system stability and universality	Sample-in-result-out integrated testing; point-of-care testing
Mass spectrometry	Bacteria, fungi, biomolecules, and metabolites	Characteristic fingerprinting based on mass-to-charge ratio differences for rapid identification and typing	Colony-dependent	Minutes after isolation	High	High	Colony-based	Fast analysis; relatively high accuracy; no need for target-specific primers; suitable for rapid confirmation	High instrument cost; complex pretreatment; discrimination of closely related species depends on database quality	Rapid species identification; outbreak investigation; high-throughput laboratory screening
Sequencing technologies	Bacteria, viruses, fungi, resistance genes, and metagenomic samples	Pathogen identification, typing, tracing, and community analysis through nucleic acid sequencing	Depth-dependent	Hours to days	High data throughput	High	Real-sample datasets	High resolution; able to discover unknown pathogens; suitable for evolutionary and source-tracing analysis	High cost; complex data analysis; standardization still needs improvement	Outbreak investigation; molecular source tracing; resistance and virulence analysis
AI-assisted analysis and big-data early warning	Images, mass spectra, sensor signals, sequencing datasets, and supply-chain information	Machine learning and deep learning for signal recognition, classification, and risk prediction	Data-dependent	Seconds to minutes after data acquisition	High	Variable	Dataset-based	Automated interpretation; strong anti-noise capability; supports risk early warning and trend analysis	Highly dependent on high-quality datasets; model interpretability and generalizability still need improvement	Image-based result interpretation; smartphone colorimetric readout; automated colony counting; risk prediction.

The values for LOD, assay time, throughput, and cost are summarized as representative ranges or qualitative estimates from reported studies. These values may vary with pathogen species, food matrix, sample pretreatment, enrichment strategy, signal readout, and validation design. Validation level indicates the main validation status reported for representative studies, such as standardized methods, artificially spiked samples, naturally contaminated samples, enriched samples, or dataset-based validation. Adapted from [8,9,10,11,12,13,14,15]. LOD, limit of detection; ELISA, enzyme-linked immunosorbent assay; LFIA, lateral flow immunoassay; IMS, immunomagnetic separation; PCR, polymerase chain reaction; LAMP, loop-mediated isothermal amplification; RPA, recombinase polymerase amplification; CRISPR, clustered regularly interspaced short palindromic repeats.

**Table 2:** Selected biosensor-based platforms for foodborne pathogen detection.

Sensor Type	Target Pathogen	Sample Type	Detection Mode	Transducing Substrate/Electrode	Limit of Detection	Linear Range	Representative References
Electrochemical biosensor	*E. coli* O157:H7	Drinking water, milk, and lettuce	DPV + EIS	Glassy carbon electrode (GCE)	7 CFU/mL	10^1^–10^8^ CFU/mL	[95]
Electrochemical biosensor	*S. aureus*	Water	SWV	Glassy carbon electrode (GCE)	1 CFU/mL	10^1^–10^6^ CFU/mL	[96]
Electrochemical biosensor	*E. coli*, *S.* Typhimurium, and *P. aeruginosa*	Drinking water, milk, serum	CV + DPV	Disposable paper-based screen-printed carbon electrode (SPCE)	1 CFU/mL	10^1^–10^6^ CFU/mL	[97]
Fluorescence sensor	*B. cereus*	Milk, rice, chicken, eggs	Fluorescence	MOF nanomaterial	4 CFU/mL	2 × 10^1^–2 × 10^8^ CFU/mL	[98]
Fluorescence sensor	*S. aureus*	Eggs	Fluorescence	Eu-MOF fluorescent nanomaterial	3 CFU/mL	7.9–7.9 × 10^8^ CFU/mL	[99]
Colorimetric sensor	*S. aureus*	Milk, serum	Colorimetry	Au–Ag alloy nanorods	25 CFU/mL	10^1^–10^6^ CFU/mL	[100]
Colorimetric sensor	*Salmonella* spp.	Milk	Colorimetry	Au@PtNPs-MBs (with H_2_O_2_–TMB colorimetric system)	89 CFU/mL	10^2^–10^6^ CFU/mL	[101]
SERS sensor	*E. coli* O157:H7	Water, milk	SERS	AuNPs	244 CFU/mL	10^3^–10^7^ CFU/mL	[102]
SERS sensor	*E. coli*, *S. aureus*, and *B. cereus*	Milk, eggs, and vegetables	SERS	Ag-pS	*E. coli*: 5 CFU/mL; *S. aureus*: 5 CFU/mL; B. cereus: 4 CFU/mL	10^1^–10^6^ CFU/mL	[103]
SPR/LSPR sensor	*S. aureus*	Milk, drinking water	SPR	D-shaped/tapered optical fiber	1.14 CFU/mL	10^2^–10^8^ CFU/mL	[104]
SPR/LSPR sensor	*S. aureus*, *E. coli*, and *S.* Typhimurium	Milk, juice	SPR	SMF	10^2^ CFU/mL	10^1^–10^6^ CFU/mL	[105]
Mass-sensitive sensor (QCM/SAW)	*E. coli* O157:H7	Milk, burgers, dumplings	QCM	Gold-coated quartz crystal microbalance	7.5 × 10^2^ CFU/mL	10^3^–10^7^ CFU/mL	[106]
Mass-sensitive sensor (QCM/SAW)	*C. jejuni*	Chicken carcass rinse and turkey mince	QCM	Gold-coated quartz crystal microbalance electrode	20–30 CFU/mL	10^1^–10^6^ CFU/mL	[107]
Mass-sensitive sensor (QCM/SAW)	*S. aureus*	NR	Love-mode SAW	ZnO-SiO2 waveguide layer	12 pmol/L	0–10 nmol/L	[93]
Mass-sensitive sensor (QCM/SAW)	*E. coli* O157:H7	Food and water	SAW	125-μm-thick PEN plastic film	6.54 × 10^5^ CFU/mL	10^6^–10^8^ CFU/mL	[94]
NanoMIP-based electrochemical and thermal biosensor	Human norovirus virus-like particles (NoV-LPs)	Romaine lettuce	Electrochemical impedance/thermal sensing	NanoMIP-modified electrodes/thermal sensor	EIS: 3.4 pg/mL; HTM: 6.5 pg/mL	Not specified	[108]
Electrochemical microfluidic aptasensor	*Cryptosporidium parvum*	Buffer, stool, and tap water	Electrochemical detection	Aptamer-functionalized hierarchical 3D gold nano-/microislands	buffer: 5 oocysts/mL; stool and tap water: 10 oocysts/mL	10–100,000 oocysts/mL	[109]
CRISPR/Cas12a-mediated click immunoassay biosensor	*Trichinella spiralis*	Pork	CRISPR-assisted fluorescence	AuNP–antibody–ssDNA probes; CuAAC reaction	0.35 ng/mL	3.125–100 ng/mL	[110]

Note: Entries are grouped by sensor type for cross-platform comparison. Because different platforms use different target analytes and signal readout formats, the units for limit of detection and linear range are retained as reported in the original references.

## Data Availability

No new data were created or analyzed in this study.
